# Factors Related to the Recurrence of Sickness Absence Due to Common Mental Health Disorders: A Systematic Review

**DOI:** 10.1007/s10926-024-10224-9

**Published:** 2024-07-10

**Authors:** Lydia in‘t Hout, Suzanne G. M. van Hees, Emma Vossen, Shirley Oomens, Dike van de Mheen, Roland W. B. Blonk

**Affiliations:** 1 Tranzo, Scientific Center for Care and Wellbeing, Tilburg School of Social and Behavioral Sciences, Tilburg University, Tilburg, The Netherlands; 2 Occupational and Health Research Group, HAN University of Applied Sciences, Nijmegen, The Netherlands; 3 Department of Primary and Community Care, Radboudumc, Nijmegen School of Occupational Health, Nijmegen, The Netherlands; 4 Department of Human Resource Studies, Tilburg School of Social and Behavioral Science, Tilburg University, Tilburg, The Netherlands; 5 Optentia, North-West University, Vanderbijlpark, South Africa

**Keywords:** Recurrent sickness absence, Common mental disorders, Prognostic factors, Occupational health, Systematic review

## Abstract

**Purpose:**

Employees who experience sickness absence (SA) due to common mental disorders (CMD) are at increased risk of recurrent sickness absence (RSA). This systematic literature review examines the factors at different levels in the work and non-work context that increase or decrease the likelihood of RSA due to CMD. The resulting knowledge enables more accurate identification of employees at risk of RSA.

**Methods:**

We conducted a search in June 2023 using the following databases: PubMed, PsycInfo, Web of Science, Cumulative Index to Nursing & Allied Health Literature (Cinahl), Embase and Business Source Ultimate (BSU). Inclusion criteria were as follows: (self-)employees, CMD, related factors, RSA. The quality of the studies was assessed using the Mixed Methods Appraisal Tool (MMAT). The Individual, Group, Leader, Organisation and Overarching/social context (IGLOO) model were used to cluster the found factors and these factors were graded by evidence grading.

**Results:**

Nineteen quantitative and one qualitative studies of mainly high and some moderate quality were included in this review. A total of 78 factors were found. These factors were grouped according to the IGLOO levels and merged in 17 key factors. After evidence grading, we found that mainly low socioeconomic status (SES) and the type of previous SA (short-term SA and SA due to CMD) are predictors of an increased risk of RSA.

**Conclusions:**

Having a low SES and previous experience of SA (short term, or due to CMD) are factors that predict the chance of RSA, implying the need for prolonged support from occupational health professionals after the employee has returned to work.

**Supplementary Information:**

The online version contains supplementary material available at 10.1007/s10926-024-10224-9.

## Introduction

Mental health problems are common in most member countries of the Organisation for Economic Co-operation and Development (OECD). Of the working-age population in these countries, 20% suffers from these problems once in their lives, and they are 30%-50% more likely to have sickness absence (SA) in their working life [1]. By SA, we mean that an employee does not appear at work due to illness or complaints. SA due to common mental disorders (CMD) has increased in most Western countries in recent decades. In this study, CMD refers to depression, anxiety disorders, adjustment disorders and stress-related disorders [2, 3]. It is currently the leading cause of SA and long-term work disability [4–7]. Previous research has demonstrated that there are significant differences in the incidence of SA between countries [8, 9], that women have higher SA rates than men [10–12] and that SA incidence is higher for blue collar workers [13, 14]. SA has major personal implications like income losses, less job security and job satisfaction, reduced wellbeing and loss of meaning from not being able to contribute to society and socialise with colleagues [1, 4, 15]. In addition, SA results in high costs to employers and society due to productivity loss and social security programmes [15].

Of the employees who experience SA due to CMD, 20%-30% have a recurrent sickness absence (RSA). RSA occurs when an employee is again on SA due to CMD after fully returning to work. 90% of the RSAs occur within three years of the previous SA [16, 17]. The period of an RSA is usually longer than the previous period of absence. In addition, frequent SAs increase the risk of work disability [16, 17]. So, RSA causes a vicious downward spiral of longer absences, more frequent absences and a higher risk of work disability. Therefore, RSA has a major impact on the employee, the employer and society. Previous research on RSA has focused on distinct areas, for example the characteristics of RSA (e.g. incidence, duration period until RSA) and interventions in prevention of SA or in return to work [4–7, 15, 16]. Because of the high risk of RSA and its impact, a better understanding of factors related to RSA is important. Occupational health professionals often support absent employees. Insight into the prognostic factors of RSA is needed for these professionals, in order to assess whether there is a risk of RSA and provide longer-term support to employees at increased risk.

The aim of our study was to systematically review current scientific knowledge on factors related to RSA due to CMD and possible knowledge gaps.

## Methods

We used the PRISMA 2020 guideline to conduct a systematic literature review [18]. This systematic literature review is registered on PROSPERO. Study protocol details are available at https://www.crd.york.ac.uk/prospero/display_record.php?RecordID=336654. Contrary to what we described in the protocol, we used MMAT for quality assessment. Based on some of the authors' experiences, MMAT seemed more feasible. We also decided to use one rather than two models to synthesise the results more clearly.

### Search Strategy

We used EBSCOhost to search six electronic databases: PubMed, PsycInfo, Web of Science, Cinahl, Embase, and BSU. The first reviewer and two experienced librarians set up the search strings, using MeSH terms, Thesaurus terms and free text words. This was done iteratively: MeSH or Thesaurus terms found in one database were searched in the other databases or added to the free text of the other databases (see Appendix 1 for the search strings). The search was conducted in June 2022, and rerun in June 2023 to include articles published after 2021. The results were exported to EndNote for de-duplication. We used the six steps formulated by Bramer et al. [19] to perform the de-duplication.

### Eligibility Criteria

Our research aims to identify the prognostic factors of RSA due to CMD. The population prognostic factors outcome (PFO) framework was employed to develop the research question, thereby defining the inclusion criteria [20]:

P: employees or self-employed people, CMD, i.e. depression, anxiety, adjustment, and stress-related disorders (including burn-out),

F: related factors (contributing and preventive).

O: RSA.

All studies on RSA due to CMD containing authors’ empirical findings on factors related to the occurrence of an RSA were included. Book chapters, abstracts for conferences and letters to editors were excluded, because these are not peer-reviewed. Literature reviews were also excluded because they provide an overview of scientific evidence rather than describing empirical research. We included studies published since 2000, because our topic gained academic interest from the late 1990s onwards. English (as leading scientific language) and Dutch (as authors’ native language) articles were included.

### Selection of Studies

Three reviewers selected the studies in three phases. In the first phase, two reviewers (LH, SH) screened the article titles and abstracts. One reviewer (LH) screened all articles, and the second reviewer (SH) screened 25% of the results. Titles and abstracts were screened using the systematic review application Rayyan [21]. In the second phase, two reviewers (LH, EV) screened the full text. One reviewer (LH) screened all articles, and the other reviewer (EV) screened 25% of the results. Disagreements in both phases were discussed and resolved by the two reviewers (7% were discussed in phase one, 16% in phase two). Cases causing disagreement or doubt were discussed within the research team (2% of articles for full text screening). In the third phase, references of the included articles were screened by one reviewer (LH) for additional relevant articles.

### Quality Assessment

We assessed the quality of the included articles, using the Mixed Methods Appraisal Tool (MMAT) [22]. This tool assesses the reliability and relevance of published articles of various study designs. One reviewer (LH) assessed all included articles, one reviewer (SH) screened 75% of the articles and one reviewer (EV) screened 25% of the articles (see Appendix 2 for the quality assessment). Disagreements were discussed and resolved by the two reviewers.

### Data Analysis

For the synthesis of the results, we used descriptive synthesis and evidence grading, based on Jansen et al. [23]. The descriptive synthesis comprised the following steps: grouping, clustering, transforming data and tabulation.

The IGLOO model was used to group the factors [17]. This model is an integrated framework for sustainable return to work. It helps to identify the resources at five levels in the work and non-work context: Individual, Group, Leader, Organisational and Overaching/social context. The individual level includes cognitive, affective and behavioural aspects, including personality traits. Group level includes the social network, and leader level includes supporting supervisor or healthcare professional. The organisational level involves characteristics of the workplace or organisation and, in non-work contexts, local support organisations. The overarching level concerns national legislation and policy. By developing practical interventions aimed at strengthening resources at these levels, sustainable return to work and therefore the prevention of RSA can be promoted [17]. So, this model helps to consider the factors at different levels in both work and non-work contexts, providing a broad overview of areas that can be related to RSA. The factors found in our study were grouped per level of the IGLOO model. Subsequently, the grouped factors were clustered by merging the factors and dividing them into subgroups. The data were transformed by harmonising the directions of the effects of the factors. For example, tenure > 4 years was found to decrease the likelihood of RSA, and tenure < 5 years was found to increase the likelihood of RSA. This was harmonised to short tenure increases the likelihood of RSA.

Evidence grading for each factor was rated based on Dekkers-Sánchez et al. [24] in consideration of the quality assessment and using the following grading:

1. Strong evidence: three studies available finding an association in the same direction, or four studies or more available, of which more than 66% find a significant association in the same direction and no more than 25% find an opposite association.
2. Weak evidence: two studies available finding an association in the same direction, or three studies available, of which two find a significant association in the same direction and the third finds no significant association.
3. Insufficient evidence: one study available.
4. Inconsistent evidence: remaining cases.

## Results

**Fig. 1:**
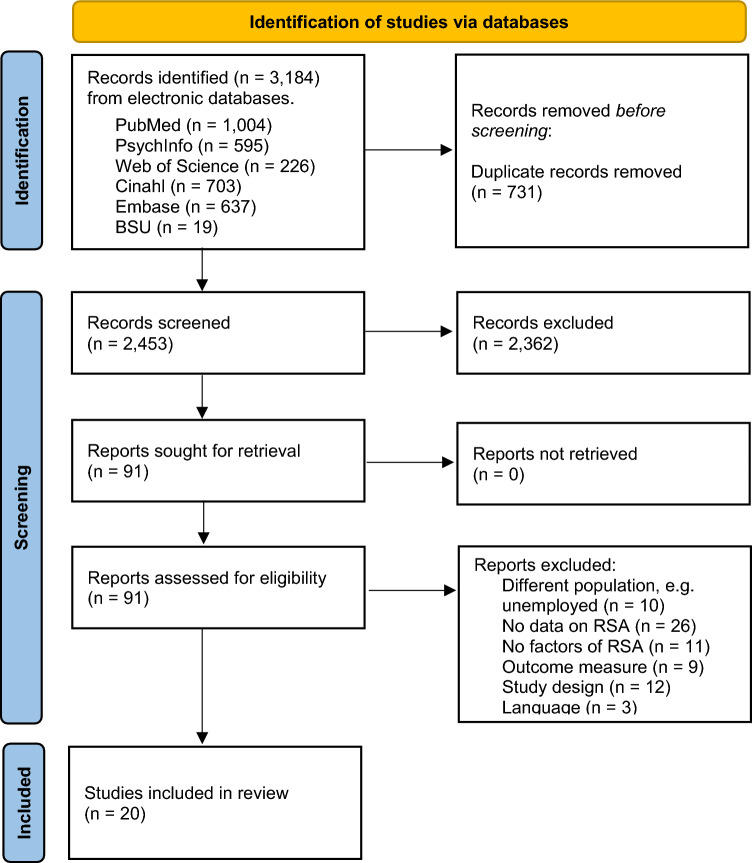
Flowchart of literature search results and inclusion/exclusion

The electronic search of the six databases identified 3,184 articles (Fig. 1). Table 1 contains an overview of the included studies. The June 2023 search yielded no new articles that fulfilled the inclusion criteria. Given the high or moderate quality of all the studies, no studies were excluded based on their quality assessment.

**Table 1:** Characteristics of the studies and found prognostic factors of RSA

	Author and year (ref)	Country	Study design	*N*	Population	Found factors related to RSA	Outcome (direction of relation)	Quality and MMAT score (high = 6–7, moderate = 4–5, low = 1–3)
1	Arends et al. 2014 [25]	Netherlands	Interventional	107	Employees with depressive, anxiety or adjustment disorders	SHARP-at work intervention	+^a^	High: 6
2	Arends et al. 2014 [26]	Netherlands	Predictive	146	Employees with depressive, anxiety or adjustment disorders	Reporting ≥ 1 chronic disease	+	High: 7
						Low supervisor social support	−^b^	
						Conflict with supervisor	−	
						Company size > 100 workers	−	
3	Arends et al. 2014 [27]	Netherlands	Interventional	131	Employees with depressive episode, recurrent depressive or persistent mood disorders	Focus on whether help was needed (in the intervention)	+	High: 7
						Focus on problems (in the intervention)	−	
4	Endo et al. 2015 [28]	Japan	Predictive	540	Employees with depression	High psychological job demands	−	High: 7
5	Ervasti et al. 2013 [29]	Finland	Predictive	125,355	Employees with depression in municipalities and hospitals	Low socioeconomic status (SES)	−	High: 7
6	Ervasti et al. 2014 [30]	Finland	Predictive	107,828	Employees with depressive episode, recurrent depressive or persistent mood disorders	Type of contract, permanent or temporary	0^c^	High: 7
7	Gaspar et al. 2018 [31]	U.S	Predictive	296,484	Employees with adjustment, bipolar or depressive disorders	Being salaried	−	Moderate: 5
						Being in a union	−	
						Previous non-CMD SA	−	
						On disability longer for the index leave	−	
						Depressive disorder	−	
						Female	−	
						Younger	−	
						Depression, uncomplicated diabetes mellitus, obesity, psychoses, and chronic pulmonary disease as comorbidity	−	
						Inpatient stays during index leave	0	
						Hourly employee	−	
						Transportation, communication, utilities industry	−	
						Point-of-service insurance coverage	−	
						Population density	−	
8	Klink, v.d. et al. 2003 [32]	Netherlands	Interventional	117	Employees with adjustment disorders	Intervention of graded activity and CBT	+	High: 6
9	Koopmans et al. 2010 [33]	Netherlands	Predictive	137,172	Employees with distress, adjustment, mild-moderate depressive or anxiety disorders	Previous SA due to CMD	−	High: 6
						Gender	0	
						Women < 35 years	−	
						Women aged 35–44	−	
						Men and age	0	
10	Koopmans et al. 2011 [16]	Netherlands	Predictive	9,904	Employees with distress symptoms, stress-related, depressive or other psychiatric disorders	Men and depressive symptoms	−	High: 7
						Women and diagnosis	0	
						Men aged 45–55	−	
						Women aged < 45	−	
						Married women	−	
						Men and marital status	0	
						Lower salary	−	
						Telecommunication company	−	
						Women and employment duration < 5 years	−	
11	Mather et al. 2019 [34]	Sweden	Predictive	2,202	Twins, with one of them experiencing SA due to depressive, anxiety, stress-related or other mental disorders	SA due to mental disorders	−	High: 6
						Familial factors	0	
12	Mattila-Holappa 2017 [35]	Finland	Predictive	13,716	Public sector employees with depressive, neurotic, stress-related, somatoform or other mental disorders	Employees aged > 34	−	High: 7
						Employees aged < 51 and low education	−	
						Employees aged < 34 and low occupational position	−	
13	Norder 2012 [36]	Europe, North America	Predictive	36	Employees with depressive episodes, recurrent depressive or persistent mood disorders	Work dysfunction	−	High: 6
						Stressful work events	−	
						Commitment to work	−	
						Number of episodes throughout lifetime	−	
						Substance abuse	−	
						Social dysfunction	−	
						DSM axis II personality disorders	−	
						Residual symptoms	−	
						DSM axis I psychopathology	−	
						Anxiety	−	
						Neuroticism	−	
						Stressful life events	−	
						Severity of first episode	−	
						Severity of last episode	−	
						Duration of last episode	−	
						Duration of first episode	−	
						Age at first episode	−	
						High demands–low control	−	
						Effort–reward imbalance	−	
						Decision latitude	−	
						Psychological job demands	−	
14	Norder et al. 2015 [37]	Netherlands	Predictive	14,369	Steel company employees with emotional disturbance, mental or behavioural disorders	Aged > 55	−	High: 6
						Production workers with emotional disturbance (vs office workers)	−	
15	Norder et al. 2015 [38]	Netherlands	Predictive	5862	Production workers with emotional disturbance, mental or behavioural disorders	Shift work (vs day work)	0	High: 6
16	Norder et al. 2015 [39]	Netherlands	Predictive	15,461	Steel company workers with emotional disturbance, mental or behavioural disorders	ICD-10 diagnosis	0	High: 7
17	Real et al. 2016 [40]	Spain	Predictive	7,112	Employees with substance dependence, affective, anxiety, adaptive, personality or other mental disorders	Short-term SA	−	High: 6
						SA that ended due to improvement (vs administrative or disability)	−	
						Public administration and transport sector	−	
						Aged 30–50 and covered by general scheme	−	
						Aged < 31 and self-employed	−	
18	Rhenen, v. et al. 2007 [41]	Netherlands	Interventional	242	Stressed and non-stressed telecom company employees	Stressed employees and cognitive intervention	−	Moderate: 4
19	Roelen et al. 2010 [42]	Netherlands	Predictive	137,172	Post en telecom company employees with mental or behavioural disorders	Higher salary scales	+	High: 7
						> 4 years of employment	+	
						Unskilled and short duration of employment	−	
20	Virtanen et al. 2011 [43]	Finland	Predictive	141,917	Employees with mania, bipolar affective, depressive, anxiety, adjustment disorders, reaction to severe stress, disorders of adult personality and behaviour, schizophrenia, schizotypal, delusional disorders, or mental and behavioural disorders owing to psychoactive substance use	Manual and lower non-manual workers	−	High: 6

^a^the factor decreases the likelihood of RSA

^b^the factor increases the likelihood of RSA

^c^The factor had no effect on RSA

We identified 78 unique factors from the 20 studies. First, these factors were grouped according to the IGLOO levels: Individual, Group, Leader, Organisation, and Overarching. The classification of some factors was not straightforward. For example, because marital status is not a true indicator of social networks, it was classified at the individual level rather than the group level. Additionally, SES is comprised of multiple indicators and the SES indicators were allocated at the individual level in both work and non-work contexts. Factors determined by the employer (e.g. salary) were positioned under the work context, while factors not determined by the employer (e.g. educational level) were positioned under the non-work context. Secondly, the factors were merged and divided in subgroups. Thirdly, we harmonised the factors, adjusting them where possible in the same direction of relationship with RSA. This descriptive analysis led to the following 17 key factors: SES, previous (type of) SA, functioning and perceptions at work, tenure, gender, comorbidity, CMD diagnosis, remaining health factors, age, functioning in private situation, contact with supervisor, company size, working conditions, cognitive interventions (provided by occupational health professionals), contract, sector and remaining factors. These results are shown in Table 2.

**Table 2:** Key factors found related to RSA

Level	Work context	Non-work context
Individual	SES: Lower salary; Employees aged < 34 and low occupational position; Unskilled and short duration of employment; Manual and lower non-manual workers	SES: Low SES; Employees aged < 51 and low education
	Previous SA: Previous non-CMD SA; On disability longer for the index leave; Previous SA due to CMD; Short-term SA; SA that ended due to improvement	Comorbidity: Reporting one or more chronic diseases; Depression, uncomplicated diabetes mellitus, obesity, psychoses, and chronic pulmonary disease as comorbidity; DSM axis II personality disorders as comorbidity; DSM axis I psychopathology as comorbidity; Anxiety as comorbidity
	Functioning and perceptions at work: Commitment to work; Work dysfunction; Stressful work events; Focus on whether help was needed in an intervention; Focus on problems in an intervention	CMD diagnose: Depressive disorder; Men and depressive symptoms; Production workers with emotional disturbance
	Tenure: Women with employment duration < 5 years; > 4 years of employment	Remaining health factors: Number of episodes of depression throughout lifetime; Residual symptoms; Severity of first episode; Severity of last episode; Duration of last episode; Duration of first episode; Age at first episode
		Functioning in private situation: Substance abuse; Social dysfunction; Stressful life events; Neuroticism
		Age: Younger; Women < 45 years; Men aged 45–55; Employees aged > 34; Aged > 55
		Gender: Female
		Remaining factors: Marital status of women
Group	No factors found	No factors found
Leader	Contact with supervisor: Low supervisor support; Conflict with supervisor	No factors found
Organisation	Company size: Company size > 100 workers	Remaining factors: Population density
	Working conditions: High psychological job demands; High demands–low control; Effort–reward imbalance; Decision latitude	
	Cognitive interventions (provided by occupational health professionals): Intervention of graded activity and CBT; Stressed employees and cognitive intervention	
	Contract: Being salaried; Hourly employee (on-call worker)	
Overarching/ social context	Sector: Transportation, (tele)communication, utilities industry; Public administration sector	No factors found
	Remaining factors: Aged < 31 and self-employed; Aged 30–50 and covered by general scheme; Point-of-service insurance; Being in a union	

### Key Factors per IGLOO Level

#### Individual Level

Four subgroups of factors were found in the work context: SES, previous SA, functioning and perceptions at work, and tenure. We found the following SES indicators in the scientific literature: education level, income level, occupational level, residence size and residence ownership. Low SES was associated with an increased risk of RSA in five studies [16, 29, 35, 42, 43]. Our findings also showed that the type of previous SA increased the likelihood of RSA, according to four studies [31, 33, 34, 40]. However, studies described this factor differently: SA due to CMD compared with other causes [31, 33, 34], the way SA ended (due to improvement, to disability or administrative ending) and short- or long-term SA [40]. Two studies described the related factors in functioning and perceptions at work: commitment to work, dysfunction, stress and focusing on problems in an intervention to improve coping increased the likelihood of RSA, while focusing on whether help was needed in that intervention decreased the likelihood of RSA [27, 36]. Finally, two studies showed that short tenure increased the risk of RSA [16, 42].

Outside the work context, multiple factors with contradictory effects were found. Five studies examined the effect of age on RSA [16, 31, 33, 35, 37], two of which examined age in interaction with gender [16, 33]. According to one study, younger age increased the risk of RSA [31], while in the remaining studies, older age increased the risk of RSA [16, 33, 35, 37]. Two studies showed that comorbidity more frequently resulted in RSA [31, 36], while one study found less RSA in case of comorbidity [26]. In terms of gender, in one study women were more likely to have RSA than men [31], while another study found no effect of gender [33]. Compared with other CMDs, two studies found that depression increased the likelihood of RSA [16, 31]. One of these studies subsequently differentiated by gender and found this to only apply for men [16]. Another study found no relation of CMD diagnosis with the risk of RSA [39]. Of the remaining factors, married women were more likely to experience RSA than unmarried women [16]. Other factors as substance abuse, neuroticism, dysfunction and stressful life events also increased the risk of RSA, according to one study. This study found seven other health-related factors that increased the likelihood of RSA [36].

The evidence grading showed two factors with strong evidence of increasing the likelihood of RSA at the individual level: type of previous SA [31, 33, 34, 40] and low SES [16, 29, 35, 42, 43]. The majority of these studies were of a high quality (eight of high and one moderate quality). Four factors had weak evidence: older age [31, 35, 37], comorbidity [26, 31, 36], depression (compared with other diagnoses) [16, 31] and short tenure [16, 42]. The evidence was inconsistent for gender [31, 33] and women aged < 45 [16, 33]. Appendix 3 presents the synthesis of the evidence grading.

#### Group Level

No factors were found at the group level.

#### Leader Level

One study found an increased risk of RSA due to low supervisor social support and conflict with the supervisor [26]. According to the evidence grading, this is insufficient evidence. No factors were reported in the non-work context.

#### Organisational Level

Most factors were found in the work context. Two studies showed that high psychological work demands, such as having to work hard and a high workload, increased the risk of RSA [28, 36]. In one of these studies, other factors in the working condition were found to increase the likelihood of RSA, like high demands–low control and effort–reward imbalance [36]. Shift work (work mornings, evenings or nights according to a shift schedule) did not affect the likelihood of RSA [38]. Two studies examined whether the contract type was related to RSA, with different results. According to one study, temporary employment had no effect on RSA [30]. Another study found that being salaried (compared to not being salaried) and being an hourly employee increased the likelihood of RSA [31]. Three studies evaluated different cognitive interventions provided by occupational health professionals that focused on improving problem-solving skills. Two of these studies showed that a cognitive intervention reduced the likelihood of RSA [25, 32], while one study found that cognitive intervention participants experienced RSA earlier than physical intervention participants [41]. Employees in companies with more than 100 workers (versus < 100) were at greater risk of RSA, according to one study [26].

In the non-work context, one study showed that population density increased the likelihood of RSA [31], without specifying whether low or high population density increased this.

According to the evidence grading, there is weak evidence that high psychological work demands [28, 36] and cognitive interventions [25, 32, 41] increase the likelihood of RSA. The evidence was inconsistent for type of contract [30, 31]. There was insufficient evidence for the remaining factors [26, 31, 36, 38, 40].

#### Overarching Level

Employees in the transportation, (tele)communication, utilities and public administration sectors (versus other sectors) were more likely to experience RSA, according to three studies [16, 31, 40]. In Spain, one study found that people aged 30–50 (compared to other age groups) contributing to social security through employment (covered by a general scheme) were more likely to experience RSA, while younger people (≤ 30) experienced RSA more often if they contributed through self-employment [40]. Point-of-service insurance (a type of managed care health insurance) and union membership showed an increased risk of RSA in another US study [31].

The evidence grading showed moderately strong evidence that working in the transport and communications sector increases the likelihood of RSA, with two high-quality studies and one moderate-quality study [16, 31, 40]. The remaining factors had insufficient evidence [31, 40].

## Discussion

We found that RSA was described differently in the studies, namely: all SA after return to work, new SA after full recovery of at least 28 days or reduction in contract hours of at least 30% regardless of full or partial return to work. For a better understanding of RSA, an internationally accepted conceptualisation of RSA should be used. Because of the increased risk of RSA due to CMD within 3 years of a previous SA, we recommend RSA to be all SA within 3 years of a full return to work after a previous SA.

This systematic review aimed to better understand RSA by identifying the factors that are related to RSA in employees with CMDs. This study shows that mainly low SES, type of previous SA and working in the transport and communications sector are related to the likelihood of RSA.

Our study shows several factors at the individual level. While previous research showed that women have higher SA rates than men [10–12], our study did not identify a clear difference between men and women in RSA. We found low SES in both work and non-work contexts. Previous research showed that people with low SES were more likely to experience mental health problems [44–46]. Employees with lower educational or occupational levels are associated with higher rates of SA [47, 48]. These findings are consistent with our results showing that low SES also increases the likelihood of RSA. However, no consensus exists in scientific research about the conceptualisation and measurement of SES [49]. Furthermore, our results do not show which dimensions of SES are related to RSA. Therefore, our results yield a tentative conclusion.

In the work context at this level, we found other related factors, including high work commitment increasing the likelihood of RSA. Previous research found that self-reported high commitment is also associated with SA due to mental disorders [50]. In the non-work context, we found that depression increased the likelihood of RSA compared to other CMDs. Previous research showed contradictory results regarding depression as a risk factor for SA. For example, one study showed that comorbid anxiety and depression as well as anxiety alone increased the likelihood of SA and was associated with RSA, while depression alone was not [51], whereas other studies showed that depressive complaints were associated with SA [52, 53]. While it is feasible that depression is more strongly related to RSA than SA, we cannot conclusively establish this based on our study.

At the leader level, our study shows that supervisor conflict and low supervisor support increases the likelihood of RSA. These findings are consistent with previous studies showing that poor supervisor relationship causes SA [54] and that supervisor support facilitates work participation and sustained return to work [23, 55, 56].

We found multiple working conditions at the organisational level that increased the likelihood of RSA. This is consistent with SA research findings showing that high work pressure causes SA [54]. Other studies have found that high efforts with low rewards [50], high job demands and low job control [48, 50] are risk factors for SA in people with CMD. Working conditions therefore appear to affect both SA and RSA.

At the overarching level, our results show that working in the transportation, (tele)communication, utilities and public administration sector increase the likelihood of RSA. Most of our findings contradict research on factors related to SA, which showed that public sector employment increased the risk of SA [57, 58].

Multiple same factors were identified in research addressing SA as in the present study, although the results are not consistent in all cases. The processes between a previous SA and RSA remain unknown and need further research.

According to the evidence grading, in half of the factors, we found insufficient or inconsistent evidence of a relationship. The differences in methodology and operationalisations of the included studies hampered comparisons. For example, we found strong evidence that employees in the transport and (tele)communication sectors are at greater risk of RSA. However, one of these studies compared a postal and a telecommunications organisation without comparing other sectors [16], which we consider insufficient evidence. Nevertheless, our study shows that previous SA and low SES are important factors negatively associated with RSA. SA is an important inclusion criterion in our research. Therefore, the finding of previous SA as a risk factor seems obvious. However, our results show that mainly the type of previous SA (namely short-term) and the cause of the SA or its ending are prognostic factors of RSA.

SA, sustainable return to work and not experiencing RSA (i.e. staying at work), is a complex process [56, 59, 60] and can be influenced by the factors such as demographic, behavioural, personal or organisational [59]. These factors, corresponding to factors we found using the IGLOO model, may be interrelated. For example, our study showed that low SES and working conditions like effort–reward imbalance and low decision latitude increased the likelihood of RSA. Employees with low SES are more likely to experience this at work [44]. Furthermore, we found that previous SA due to CMD increased the likelihood of RSA. Low SES by itself appears to increase the likelihood of CMDs [44], which in turn, according to our findings, increases the likelihood of RSA. Therefore, it is possible that these factors intermediate.

Some of the studies in our research did indeed show interrelations between factors, such as gender and age [16, 33], age and occupational position [35] or gender and marital status [16]. However, the studies have different focuses and the researchers correct for different variables. Although we cannot draw decisive conclusions about the interrelations between factors, our results provide occupational health professionals some guidance in identifying employees at increased risk of RSA, i.e. employees with a lower SES and employees who have experienced previous SA (short-term or due to CMD), in order to determine who needs prolonged support.

## Strengths and Limitations

To the best of our knowledge, this is the first systematic review on this topic. This study provides an important insight into the scientific knowledge on factors related to RSA. Previous research is limited, and by conducting an extensive search and synthesising the results, an up-to-date overview of current scientific knowledge has emerged with results up to June 2023.

Strengths of our study are that we synthesised the data using descriptive synthesis and evidence grading. Furthermore, we included studies with different methodologies. Although we can only draw tentative conclusions, this provides an initial overview of the prognostic factors for RSA that have been identified in the scientific literature. Another strength of this study is the grouping of the identified factors according to a framework used for sustainable return to work, namely the IGLOO model. This provides a comprehensive overview of related factors at different levels related to the employee, considering both work and non-work contexts.

This study also has some limitations. First, in grading the evidence, we combined the results of studies with different aims and methodologies. This means that conclusions about the strength of the evidence should be made with caution. Furthermore, while cultural differences may also influence RSA, our results are based mainly on studies from Western countries. Therefore, relevant information from other cultures may be missing. The study is also limited by the selection on language and the small number of articles found.

## Implications for Practice and Future Research

This study showed that low SES and the type of SA are important factors related to RSA. In practice, therefore, it is important to support low SES employees after their return to work, in order to prevent RSA. Occupational health professionals can contribute by advising employers and extending their support to the employee after SA. They can focus on modifiable factors associated with SES, such as working conditions (which are often worse in case of low SES [44]) or finances. Extending support after returning to work also concerns employees with pervious short-term SA or previous SA due to CMD.

Relatively little is known regarding RSA, and the current scientific knowledge is mainly based on quantitative research using pre-existing registries. Factors that are more difficult to assess quantitatively, like interpersonal relationships, support, perceptions and experiences, may also relate to RSA. Previous research on return to work and stay at work used qualitative or mixed methods to identify the related factors [61–63]. Research using these methods is important to help understand and prevent RSA. Qualitative research among employees experiencing RSA due to CMD is necessary to reveal the complexities and processes affecting RSA.

## Conclusion

We synthesised the results of 20 studies on factors related to RSA due to CMD. This study showed that mainly low SES and the type of previous SA (short-term SA or SA due to CMD) are related to RSA. Support from occupational health professionals for employees with low SES and/or who have experienced previous SA (short-term SA, SA due to CMD) should therefore be extended after their return to work, with long-term monitoring and discussion of barriers to work.

## Supplementary Information

Below is the link to the electronic supplementary material.

Supplementary file1 (DOCX 65 KB)

## Data Availability

No datasets were generated or analysed during the current study.
