# Supplementary material for: Factors Related to the Recurrence of Sickness Absence Due to Common Mental Health Disorders: A Systematic Review
Source: J Occup Rehabil. 2024 Jul 10;35(3):435–49. doi: 10.1007/s10926-024-10224-9 (PMC12361277; doi:10.1007/s10926-024-10224-9)
Supplement: Supplementary file 1 — Supplementary file1 (DOCX 65 KB) [file 10926_2024_10224_MOESM1_ESM.docx]

**Factors related to the recurrence of sickness absence due to common mental health disorders: a systematic reviewˡ**

By Lydia in ‘t Hout, MSc², Suzanne GM van Hees, PhD, Emma Vossen, PhD, Shirley Oomens, Dr., Dike van de Mheen, Prof. Dr., Roland WB Blonk, Prof. Dr.

Journal of Occupational Rehabilitation

1. Supplementary material
2. Correspondence to: Lydia in ‘t Hout, Tranzo Scientific Center for Care and Wellbeing, P.O. Box 90153, 5000 LE Tilburg, The Netherlands. E-mail: l.inthout@tilburguniversity.edu.

**Appendix 1: searchstrings**

**PubMed**

*S1. Influencing factors*

"Risk Factors"[Mesh] OR "Risk"[Mesh] OR "Probability"[Mesh] OR "Protective Factors"[Mesh] OR "Risk Assessment"[Mesh] OR "Risk Adjustment"[Mesh] OR "Risk Management"[Mesh] OR "Forecasting"[Mesh] OR "Prognosis"[Mesh] OR "Causality"[Mesh] OR predictor [tiab] OR prospective [tiab] OR prognos*[tiab] OR Risk factor*[tiab] OR Protective factor*[tiab] OR Influence factor*[tiab] OR Enabling factor*[tiab] OR Promoting factor*[tiab] OR risk Adjustment*[tiab] OR risk assessment*[tiab] OR risk management*[tiab]

*S2. Recidivism*

"Recurrence"[Mesh] OR "Rehabilitation"[Mesh] OR "Rehabilitation, Vocational"[Mesh] OR Recurrence [tiab] OR Relapse [tiab] OR Recidive [tiab] OR reintegration [tiab] OR job reentry [tiab] OR vocational rehabilitation [tiab] OR rehabilitation [tiab]

*S3. Absenteeism*

"Absenteeism"[Mesh] OR "Sick Leave"[Mesh] OR "Return to Work"[Mesh] OR Absenteeism [tiab] OR return to work [tiab] OR Work capacity [tiab] OR Work disability [tiab] OR work incapacity [tiab] OR work incapability [tiab] OR work inhibition [tiab] OR medical leave [tiab] OR disability leave [tiab] OR sick leave [tiab] OR absente* [tiab] OR work absence [tiab] OR work presence [tiab] OR convalescen* [tiab] OR sick day [tiab] OR illness day [tiab] OR sickness absence [tiab] OR recovery of function*[tiab] OR presenteeism [tiab] OR work absenteeism [tiab] OR work day loss [tiab] OR work time loss [tiab] OR work participation [tiab] OR occupational health [tiab]

*S4. Common mental health*

“Persons with Mental Disabilities"[Mesh] OR "Disabled Persons"[Mesh] OR "Mental Health"[Mesh] OR "Mental Disorders"[Mesh] OR Common mental health [tiab] OR Common mental disorder* [tiab] OR Common mental illness* [tiab] OR Common mental diseas* [tiab] OR mental suffering [tiab]

*S5. Depression*

"Depression"[Mesh] OR "Dysthymic Disorder"[Mesh] OR "Seasonal Affective Disorder"[Mesh] OR "Depressive Disorder, Treatment-Resistant"[Mesh] OR "Depressive Disorder, Major"[Mesh] OR "Depressive Disorder"[Mesh] OR Depress* [tiab] OR Melanchol* [tiab]

*S6. Anxiety*

"Anxiety"[Mesh] OR "Anxiety Disorders"[Mesh] OR "Phobia, Social"[Mesh] OR "Fear"[Mesh] OR "Panic"[Mesh] OR "Phobic Disorders"[Mesh] OR "Neurotic Disorders"[Mesh] OR "Obsessive-Compulsive Disorder"[Mesh] OR "Psychological Distress"[Mesh] OR anxiet* [tiab] OR anxious* [tiab] OR phobi* [tiab] OR Panic disorder* [tiab] OR Panic attack* [tiab] OR fear* [tiab] OR scared [tiab] OR afraid [tiab] OR Worrie* [tiab] OR worry [tiab] OR Neuros* [tiab] OR Neurotic* [tiab] OR Angst [tiab] OR apprehension [tiab] OR disquiet* [tiab] OR dread* [tiab] OR terrify [tiab] OR terrified* [tiab] OR nervousness [tiab] OR jitter* [tiab] OR restlessness* [tiab] OR Neurotic Disorder* [tiab] OR Psychoneurose* [tiab] OR Obsessive Compulsive Neuros* [tiab] OR Obsessive compulsive disorder* [tiab] OR Panic* [tiab] OR Claustrophobia* [tiab] OR Negative feeling* [tiab] OR Negative emotion [tiab] OR Negative thought* [tiab] OR Emotional well-being [tiab] OR Emotional benefit* [tiab] OR Emotional respons* [tiab] OR antianxiet* [tiab] OR antifear* [tiab]

*S7. Stress*

"Stress, Psychological"[Mesh] OR "Stress, Physiological"[Mesh] OR "Stress Disorders, Post-Traumatic"[Mesh] OR "Stress Disorders, Traumatic, Acute"[Mesh] OR "Stress Disorders, Traumatic"[Mesh] OR "Occupational Stress"[Mesh] OR "Adjustment Disorders"[Mesh] OR "Burnout, Professional"[Mesh] OR "Burnout, Psychological"[Mesh] OR stress* [tiab] OR Nervousnes* [tiab] OR psychological distress [tiab] OR psychological adjustment [tiab] OR psychological disorder [tiab] OR mental suffer* [tiab] OR psychological suffer* [tiab] OR burnout [tiab]

*S8*

#4 OR #5 OR #6 OR #7

*S9*

#8 AND #1 AND #2 AND #3

**PsychInfo**

*S1. Influencing factors*

DE "Working Conditions" OR DE "Risk Factors" OR DE "Protective Factors" OR DE "Risk Assessment" OR TI (predictor OR prospective OR prognos* OR ((Risk* OR protective OR influenc* OR enabl* OR advancing OR promoting) N1 factor*) OR (risk N1 (Adjustment OR assessment OR management))) OR SU (predictor OR prospective OR prognos* OR ((Risk* OR protective OR influenc* OR enabl* OR advancing OR promoting) N1 factor*) OR (risk N1 (Adjustment OR assessment OR management))) OR AB (predictor OR prospective OR prognos* OR ((Risk* OR protective OR influenc* OR enabl* OR advancing OR promoting) N1 factor*) OR (risk N1 (Adjustment OR assessment OR management)))

*S2. Recidivism*

DE "Reemployment" OR DE "Relapse (Disorders)” OR DE "Relapse Prevention" OR DE "Prevention" OR DE "Rehabilitation" OR TI (recurren* OR Relapse OR Recidiv* OR reintegration OR (job N1 (reentry OR re-entry)) OR (vocational N1 rehabilitation)) OR SU ((recurren* OR Relapse OR Recidiv* OR reintegration OR (job N1 (reentry OR re-entry)) OR (vocational N1 rehabilitation)) OR AB (recurren* OR Relapse OR Recidiv* OR reintegration OR (job N1 (reentry OR re-entry)) OR (vocational N1 rehabilitation))

*S3. Absenteeism*

DE "Employee Absenteeism" OR DE "Employee Leave Benefits" OR DE "Disability Management" OR TI (((repetitive OR repeated OR patterned) N1 absent*) OR (return N1 work) OR (work* N1 capacity) OR (work* N1 disability) OR (work* N1 incapacity) OR (work* N1 incapability) OR (work* N1 inhibition) OR ((medical OR sick OR disability) N1 leave) OR absente* OR (work N1 (absence OR presence)) OR (disability N1 absence) OR convalescen* OR (sick OR illness N1 day*) OR (recovery N1 function*) OR presenteeism OR (sickness N1 absence) OR (work N1 absenteeism) OR (work N1 day N1 loss) OR (work N1 time N1 loss) OR (work N1 participation) OR (occupational N1 health)) OR AB (((repetitive OR repeated OR patterned) N1 absent*) OR (return N1 work) OR (work* N1 capacity) OR (work* N1 disability) OR (work* N1 incapacity) OR (work* N1 incapability) OR (work* N1 inhibition) OR ((medical OR sick OR disability) N1 leave) OR absente* OR (work N1 (absence OR presence)) OR (disability N1 absence) OR convalescen* OR (sick OR illness N1 day*) OR (recovery N1 function*) OR presenteeism OR (sickness N1 absence) OR (work N1 absenteeism) OR (work N1 day N1 loss) OR (work N1 time N1 loss) OR (work N1 participation) OR (occupational N1 health)) OR SU (((repetitive OR repeated OR patterned) N1 absent*) OR (return N1 work) OR (work* N1 capacity) OR (work* N1 disability) OR (work* N1 incapacity) OR (work* N1 incapability) OR (work* N1 inhibition) OR ((medical OR sick OR disability) N1 leave) OR absente* OR (work N1 (absence OR presence)) OR (disability N1 absence) OR convalescen* OR (sick OR illness N1 day*) OR (recovery N1 function*) OR presenteeism OR (sickness N1 absence) OR (work N1 absenteeism) OR (work N1 day N1 loss) OR (work N1 time N1 loss) OR (work N1 participation) OR (occupational N1 health))

*S4. Common mental health*

DE "Serious Mental Illness" OR DE "Mental Disorders" OR DE "Chronic Mental Illness" OR DE "Mental Illness (Attitudes Toward)" OR TI (Common N1 mental N1 (disorder* OR Health OR illness OR diseas* OR problem*)) OR SU (Common N1 mental N1 (disorder* OR Health OR illness OR diseas* OR problem*)) OR AB (Common N1 mental N1 (disorder* OR Health OR illness OR diseas* OR problem*))

*S5. Depression*

DE "Recurrent Depression" OR DE "Depression (Emotion)" OR DE "Treatment Resistant Depression" OR DE "Atypical Depression" OR DE "Reactive Depression" OR DE "Major Depression" OR DE "Anaclitic Depression" OR DE "Dysthymic Disorder" OR DE "Endogenous Depression" OR TI (depress* OR melanchol*) OR SU (depress* OR melanchol*) OR AB (depress* OR melanchol*)

*S6. Anxiety*

DE “Anxiety” OR DE "Anxiety Disorders" OR DE "Generalized Anxiety Disorder" OR DE "Social Anxiety" OR DE “Social Anxiety Disorder” OR TI (anxiet* OR anxious* OR phobi* OR (panic N1 (disorder* OR attack)) OR fear* OR scared OR afraid OR Worrie* OR worry OR Neuros* OR neurotic OR angst OR apprehension OR disquiet* OR dread* OR terrify OR terrified* OR fright* OR horror* OR nervousness OR jitter* OR uneas* OR restlessness* OR (Neurotic N1 Disorder*) OR Psychoneuroses OR (Obsessive N1 Compulsive N1 (Neuros* OR disorder)) OR Panic* OR Claustrophobia* OR (Mental N1 suffering) OR (Negative N1 (feeling* OR emotion OR thought*)) OR (Emotional N1 well N1 being) OR (Emotional N1 (benefit* OR respons*)) OR antianxiet* OR antifear*) OR SU (anxiet* OR anxious* OR phobi* OR (panic N1 (disorder* OR attack)) OR fear* OR scared OR afraid OR Worrie* OR worry OR Neuros* OR neurotic OR angst OR apprehension OR disquiet* OR dread* OR terrify OR terrified* OR fright* OR horror* OR nervousness OR jitter* OR uneas* OR restlessness* OR (Neurotic N1 Disorder*) OR Psychoneuroses OR (Obsessive N1 Compulsive N1 (Neuros* OR disorder)) OR Panic* OR Claustrophobia* OR (Mental N1 suffering) OR (Negative N1 (feeling* OR emotion OR thought*)) OR (Emotional N1 well N1 being) OR (Emotional N1 (benefit* OR respons*)) OR antianxiet* OR antifear*) OR AB (anxiet* OR anxious* OR phobi* OR (panic N1 (disorder* OR attack)) OR fear* OR scared OR afraid OR Worrie* OR worry OR Neuros* OR neurotic OR angst OR apprehension OR disquiet* OR dread* OR terrify OR terrified* OR fright* OR horror* OR nervousness OR jitter* OR uneas* OR restlessness* OR (Neurotic N1 Disorder*) OR Psychoneuroses OR (Obsessive N1 Compulsive N1 (Neuros* OR disorder)) OR Panic* OR Claustrophobia* OR (Mental N1 suffering) OR (Negative N1 (feeling* OR emotion OR thought*)) OR (Emotional N1 well N1 being) OR (Emotional N1 (benefit* OR respons*)) OR antianxiet* OR antifear*)

*S7. Stress*

DE "Stress and Trauma Related Disorders" OR DE "Adjustment Disorders" OR DE "Occupational Stress" OR DE "Emotional Adjustment" OR DE "Adjustment" OR DE "Adaptability (Personality)" OR DE "Chronic Stress" OR DE "Environmental Stress" OR DE "Physiological Stress" OR DE "Stress" OR DE "Social Stress" OR DE "Emotional States" OR DE "Acute Stress Disorder" OR DE "Burnout"

OR TI (stress* OR Nervousnes* OR (psychological N1 distress) OR (adjustment N1 disorder) OR ((mental OR psycholog*) N1 suffer*) OR burnout) OR SU (stress* OR Nervousnes* OR (psychological N1 distress) OR (adjustment N1 disorder) OR ((mental OR psycholog*) N1 suffer*) OR burnout) OR AB (stress* OR Nervousnes* OR (psychological N1 distress) OR (adjustment N1 disorder) OR ((mental OR psycholog*) N1 suffer*) OR burnout)

*S8*

S4 OR S5 OR S6 OR S7

*S9*

S8 AND S1 AND S2 AND S3

**Web of Science**

*S1. Influencing factors*

TS=(predictor OR prospective OR prognos* OR ((Risk* OR protective OR influenc* OR enabl* OR advancing OR promoting) NEAR/1 factor*) OR (risk NEAR/1 (Adjustment OR assessment OR management)))

*S2. Recidivism*

TS=(recurren* OR Relapse OR Recidiv* OR reintegration OR (job NEAR/1 (reentry OR re-entry)) OR (vocational NEAR/1 rehabilitation))

*S3. Absenteeism*

TS=(((repetitive OR repeated OR patterned) NEAR/1 absent*) OR (return NEAR/1 work) OR (work* NEAR/1 capacity) OR (work* NEAR/1 disability) OR (work* NEAR/1 incapacity) OR (work* NEAR/1 incapability) OR (work* NEAR/1 inhibition) OR ((medical OR sick OR disability) NEAR/1 leave) OR absente* OR (work NEAR/1 (absence OR presence)) OR (disability NEAR/1 absence) OR convalescen* OR (sick OR illness NEAR/1 day*) OR (recovery NEAR/1 function*) OR presenteeism OR (sickness NEAR/1 absence) OR (work NEAR/1 absenteeism) OR (work NEAR/1 day NEAR/1 loss) OR (work NEAR/1 time NEAR/1 loss) OR (work NEAR/1 participation) OR (occupational NEAR/1 health))

*S4. Common mental health*

TS=(Common NEAR/1 mental NEAR/1 (disorder* OR Health OR illness OR diseas* OR problem*))

*S5. Depression*

TS=(depress* OR melanchol*)

*S6. Anxiety*

TS=((psychological NEAR/1 distress) OR anxiet* OR antianxiet* OR anxious* OR phobi* OR (panic NEAR/1 (disorder* OR attack)) OR fear* OR antifear* OR scared OR afraid OR Worrie* OR worry OR Neuros* OR neurotic OR angst OR apprehension OR disquiet* OR dread* OR terrify OR terrified* OR fright* OR horror* OR nervousness OR jitter* OR uneas* OR restlessness* OR (Neurotic NEAR/1 Disorder*) OR Psychoneuroses OR (Obsessive NEAR/1 Compulsive NEAR/1 (Neuros* OR disorder)) OR Claustrophobia* OR (Mental NEAR/1 suffering) OR (Negative NEAR/1 (feeling* OR emotion OR thought*)) OR (Emotional NEAR/1 well NEAR/1 being) OR (Emotional NEAR/1 (benefit* OR respons*)))

*S7. Stress*

TS=(stress* OR Nervousnes* OR (psychological NEAR/1 distress) OR (adjustment NEAR/1 disorder) OR ((mental OR psycholog*) NEAR/1 suffer*) OR burnout)

*S8*

#4 OR #5 OR #6 OR #7

*S9*

#8 AND #1 AND #2 AND #3

**Cinahl**

*S1. Influencing factors*

MH "Risk Factors" OR MH "Work Environment" OR MH "Forecasting" OR TI (predictor OR prospective OR prognos* OR ((Risk* OR protective OR influenc* OR enabl* OR advancing OR promoting) N1 factor*) OR (risk N1 (Adjustment OR assessment OR management))) OR SU (predictor OR prospective OR prognos* OR ((Risk* OR protective OR influenc* OR enabl* OR advancing OR promoting) N1 factor*) OR (risk N1 (Adjustment OR assessment OR management))) OR AB (predictor OR prospective OR prognos* OR ((Risk* OR protective OR influenc* OR enabl* OR advancing OR promoting) N1 factor*) OR (risk N1 (Adjustment OR assessment OR management)))

*S2. Recidivism*

MH "Recurrence" OR MH "Recidivism" OR MH "Rehabilitation" OR MH "Job Re-Entry" OR TI (recurren* OR Relapse OR Recidiv* OR reintegration OR (job N1 (reentry OR re-entry)) OR (vocational N1 rehabilitation)) OR SU ((recurren* OR Relapse OR Recidiv* OR reintegration OR (job N1 (reentry OR re-entry)) OR (vocational N1 rehabilitation)) OR AB (recurren* OR Relapse OR Recidiv* OR reintegration OR (job N1 (reentry OR re-entry)) OR (vocational N1 rehabilitation))

*S3. Absenteeism*

MH "Absenteeism" OR MH "Sick Leave" OR MH "Disability Management" OR TI (((repetitive OR repeated OR patterned) N1 absent*) OR (return N1 work) OR (work* N1 capacity) OR (work* N1 disability) OR (work* N1 incapacity) OR (work* N1 incapability) OR (work* N1 inhibition) OR ((medical OR sick OR disability) N1 leave) OR absente* OR (work N1 (absence OR presence)) OR (disability N1 absence) OR convalescen* OR (sick OR illness N1 day*) OR (recovery N1 function*) OR presenteeism OR (sickness N1 absence) OR (work N1 absenteeism) OR (work N1 day N1 loss) OR (work N1 time N1 loss) OR (work N1 participation) OR (occupational N1 health)) OR AB (((repetitive OR repeated OR patterned) N1 absent*) OR (return N1 work) OR (work* N1 capacity) OR (work* N1 disability) OR (work* N1 incapacity) OR (work* N1 incapability) OR (work* N1 inhibition) OR ((medical OR sick OR disability) N1 leave) OR absente* OR (work N1 (absence OR presence)) OR (disability N1 absence) OR convalescen* OR (sick OR illness N1 day*) OR (recovery N1 function*) OR presenteeism OR (sickness N1 absence) OR (work N1 absenteeism) OR (work N1 day N1 loss) OR (work N1 time N1 loss) OR (work N1 participation) OR (occupational N1 health)) OR SU (((repetitive OR repeated OR patterned) N1 absent*) OR (return N1 work) OR (work* N1 capacity) OR (work* N1 disability) OR (work* N1 incapacity) OR (work* N1 incapability) OR (work* N1 inhibition) OR ((medical OR sick OR disability) N1 leave) OR absente* OR (work N1 (absence OR presence)) OR (disability N1 absence) OR convalescen* OR (sick OR illness N1 day*) OR (recovery N1 function*) OR presenteeism OR (sickness N1 absence) OR (work N1 absenteeism) OR (work N1 day N1 loss) OR (work N1 time N1 loss) OR (work N1 participation) OR (occupational N1 health))

*S4. Common mental health*

MH "Mentally Disabled Persons" OR MH "Mental Health" OR MH "Mental Disorders" OR MH "Mental Disorders, Chronic" OR MH "Attitude to Mental Illness" OR TI (Common N1 mental N1 (disorder* OR Health OR illness OR diseas* OR problem*)) OR SU (Common N1 mental N1 (disorder* OR Health OR illness OR diseas* OR problem*)) OR AB (Common N1 mental N1 (disorder* OR Health OR illness OR diseas* OR problem*))

*S5. Depression*

MH "Depression" OR MH "Emotional Lability" OR MH "Seasonal Affective Disorder" OR MH "Depression, Reactive" OR MH "Dysthymic Disorder" OR TI Depress* OR Melanchol* OR AB Depress* OR Melanchol* OR SU Depress* OR Melanchol*

*S6. Anxiety*

MH "Anxiety" OR MH "Fear" OR MH "Generalized Anxiety Disorder" OR MH "Social Anxiety Disorders" OR MH "Anxiety Disorders" OR MH "Anticipatory Anxiety" OR MH "Phobic Disorders" OR MH "Panic Disorder" OR TI (anxiet* OR anxious* OR phobi* OR (panic N1 (disorder* OR attack)) OR fear* OR scared OR afraid OR Worrie* OR worry OR Neuros* OR neurotic OR angst OR apprehension OR disquiet* OR dread* OR terrify OR terrified* OR fright* OR horror* OR nervousness OR jitter* OR uneas* OR restlessness* OR (Neurotic N1 Disorder*) OR Psychoneuroses OR (Obsessive N1 Compulsive N1 (Neuros* OR disorder)) OR Panic* OR Claustrophobia* OR (Mental N1 suffering) OR (Negative N1 (feeling* OR emotion OR thought*)) OR (Emotional N1 well N1 being) OR (Emotional N1 (benefit* OR respons*)) OR antianxiet* OR antifear*) OR SU (anxiet* OR anxious* OR phobi* OR (panic N1 (disorder* OR attack)) OR fear* OR scared OR afraid OR Worrie* OR worry OR Neuros* OR neurotic OR angst OR apprehension OR disquiet* OR dread* OR terrify OR terrified* OR fright* OR horror* OR nervousness OR jitter* OR uneas* OR restlessness* OR (Neurotic N1 Disorder*) OR Psychoneuroses OR (Obsessive N1 Compulsive N1 (Neuros* OR disorder)) OR Panic* OR Claustrophobia* OR (Mental N1 suffering) OR (Negative N1 (feeling* OR emotion OR thought*)) OR (Emotional N1 well N1 being) OR (Emotional N1 (benefit* OR respons*)) OR antianxiet* OR antifear*) OR AB (anxiet* OR anxious* OR phobi* OR (panic N1 (disorder* OR attack)) OR fear* OR scared OR afraid OR Worrie* OR worry OR Neuros* OR neurotic OR angst OR apprehension OR disquiet* OR dread* OR terrify OR terrified* OR fright* OR horror* OR nervousness OR jitter* OR uneas* OR restlessness* OR (Neurotic N1 Disorder*) OR Psychoneuroses OR (Obsessive N1 Compulsive N1 (Neuros* OR disorder)) OR Panic* OR Claustrophobia* OR (Mental N1 suffering) OR (Negative N1 (feeling* OR emotion OR thought*)) OR (Emotional N1 well N1 being) OR (Emotional N1 (benefit* OR respons*)) OR antianxiet* OR antifear*)

*S7. Stress*

MH "Psychological Distress" OR MH "Stress Disorders, Post-Traumatic" OR MH "Adjustment Disorders" OR MH "Stress, Occupational" OR MH "Burnout, Professional" OR MH "Stress, Physiological" OR TI (stress* OR Nervousnes* OR (psychological N1 distress) OR (adjustment N1 disorder) OR ((mental OR psycholog*) N1 suffer*) OR burnout) OR AB (stress* OR Nervousnes* OR (psychological N1 distress) OR (adjustment N1 disorder) OR ((mental OR psycholog*) N1 suffer*) OR burnout) OR SU (stress* OR Nervousnes* OR (psychological N1 distress) OR (adjustment N1 disorder) OR ((mental OR psycholog*) N1 suffer*) OR burnout)

*S8*

S4 OR S5 OR S6 OR S7

*S9*

S8 AND S1 AND S2 AND S3

**Embase**

*S1. Influencing factors*

'risk'/de OR 'prediction and forecasting'/de OR 'prognosis'/de OR 'risk factor'/de OR 'predictor variable'/exp OR (predictor OR prospective OR prognos* OR ((Risk* OR protective OR influenc* OR enabl* OR advancing OR promoting) NEAR/1 factor*) OR (risk NEAR/1 (Adjustment OR assessment OR management))):ti,ab,kw

*S2. Recidivism*

'work resumption'/de OR 'vocational rehabilitation'/de OR 'recidivism'/de OR 'recurrence risk'/de OR (recurren* OR Relapse OR Recidiv* OR reintegration OR (job NEAR/1 (reentry OR re-entry)) OR (vocational NEAR/1 rehabilitation)):ti,ab,kw

*S3. Absenteeism*

'presenteeism'/de OR 'medical leave'/de OR 'work capacity'/de OR 'work disability'/de OR 'absenteeism'/de OR (((repetitive OR repeated OR patterned) NEAR/1 absent*) OR (return NEAR/1 work) OR (work* NEAR/1 capacity) OR (work* NEAR/1 disability) OR (work* NEAR/1 incapacity) OR (work* NEAR/1 incapability) OR (work* NEAR/1 inhibition) OR ((medical OR sick OR disability) NEAR/1 leave) OR absente* OR (work NEAR/1 (absence OR presence)) OR (disability NEAR/1 absence) OR convalescen* OR (sick OR illness NEAR/1 day*) OR (recovery NEAR/1 function*) OR presenteeism OR (sickness NEAR/1 absence) OR (work NEAR/1 absenteeism) OR (work NEAR/1 day NEAR/1 loss) OR (work NEAR/1 time NEAR/1 loss) OR (work NEAR/1 participation) OR (occupational NEAR/1 health)):ti,ab,kw

*S4. Common mental health*

'mental health'/de OR 'mental disease'/de OR (Common NEAR/1 mental NEAR/1 (disorder* OR Health OR illness OR diseas* OR problem*) OR (Emotional NEAR/1 well NEAR/1 being) OR (Emotional NEAR/1 benefit*) OR (Emotional NEAR/1 respons*)):ti,ab,kw

*S5. Depression*

'melancholia'/de OR 'depression'/de OR 'dysthymia'/de OR 'atypical depression'/de OR 'chronic depression'/de OR 'recurrent brief depression'/de OR 'treatment resistant depression'/de OR 'reactive depression'/de OR 'endogenous depression'/de OR 'major depression'/de OR 'minor depression'/de OR (depress* OR melanchol*):ti,ab,kw

*S6. Anxiety*

'anxiety'/de OR 'anxiety disorder'/de OR 'distress syndrome'/de OR 'generalized anxiety disorder'/de OR 'social anxiety'/de OR ((psychological NEAR/1 distress) OR anxiet* OR antianxiet* OR anxious* OR phobi* OR (panic NEAR/1 (disorder* OR attack)) OR fear* OR antifear* OR scared OR afraid OR Worrie* OR worry OR Neuros* OR neurotic OR angst OR apprehension OR disquiet* OR dread* OR terrify OR terrified* OR fright* OR horror* OR nervousness OR jitter* OR uneas* OR restlessness* OR (Neurotic NEAR/1 Disorder*) OR Psychoneuroses OR (Obsessive NEAR/1 Compulsive NEAR/1 (Neuros* OR disorder)) OR Claustrophobia* OR (Mental NEAR/1 suffering) OR (Negative NEAR/1 (feeling* OR emotion OR thought*)) OR (Emotional NEAR/1 well NEAR/1 being) OR (Emotional NEAR/1 (benefit* OR respons*))):ti,ab,kw

*S7. Stress*

'social stress'/de OR 'job stress'/de OR 'environmental stress'/de OR 'physiological stress'/de OR 'mental stress'/de OR 'burnout'/de OR 'adjustment disorder'/de OR chronic stress'/de OR 'acute stress'/de OR (stress* OR Nervousnes* OR (adjustment NEAR/1 disorder) OR ((mental OR psycholog*) NEAR/1 suffer*) OR burnout):ti,ab,kw

*S8*

#4 OR #5 OR #6 OR #7

*S9*

#8 AND #1 AND #2 AND #3

**Business Source Ultimate**

*S1. Influencing factors*

DE "WORK environment" OR DE "FORECASTING" OR TI (predictor OR prospective OR prognos* OR ((Risk* OR protective OR influenc* OR enabl* OR advancing OR promoting) N1 factor*) OR (risk N1 (Adjustment OR assessment OR management))) OR SU (predictor OR prospective OR prognos* OR ((Risk* OR protective OR influenc* OR enabl* OR advancing OR promoting) N1 factor*) OR (risk N1 (Adjustment OR assessment OR management))) OR AB (predictor OR prospective OR prognos* OR ((Risk* OR protective OR influenc* OR enabl* OR advancing OR promoting) N1 factor*) OR (risk N1 (Adjustment OR assessment OR management)))

*S2. Recidivism*

DE "REHABILITATION worker attitudes" OR TI (recurren* OR Relapse OR Recidiv* OR reintegration OR (job N1 (reentry OR re-entry)) OR (vocational N1 rehabilitation)) OR SU ((recurren* OR Relapse OR Recidiv* OR reintegration OR (job N1 (reentry OR re-entry)) OR (vocational N1 rehabilitation)) OR AB (recurren* OR Relapse OR Recidiv* OR reintegration OR (job N1 (reentry OR re-entry)) OR (vocational N1 rehabilitation))

*S3. Absenteeism*

DE "SICK leave" OR DE "ATTENDANCE" OR DE "JOB absenteeism" OR DE "EMPLOYMENT reentry" OR DE "VOCATIONAL rehabilitation" OR DE "RETURN to work programs" OR DE "PRESENTEEISM (Labor)" OR TI (((repetitive OR repeated OR patterned) N1 absent*) OR (return N1 work) OR (work* N1 capacity) OR (work* N1 disability) OR (work* N1 incapacity) OR (work* N1 incapability) OR (work* N1 inhibition) OR ((medical OR Sick OR disability) N1 leave) OR absente* OR (work N1 (absence OR presence)) OR (disability N1 absence) OR convalescen* OR (sick N1 day*) OR (illness N1 day*) OR (recovery N1 function*) OR presenteeism OR (sickness N1 absence) OR (work N1 absenteeism) OR (work N1 day N1 loss) OR (work N1 time N1 loss) OR (work N1 participation) OR (occupational N1 health)) OR AB (((repetitive OR repeated OR patterned) N1 absent*) OR (return N1 work) OR (work* N1 capacity) OR (work* N1 disability) OR (work* N1 incapacity) OR (work* N1 incapability) OR (work* N1 inhibition) OR ((medical OR Sick OR disability) N1 leave) OR absente* OR (work N1 (absence OR presence)) OR (disability N1 absence) OR convalescen* OR (sick N1 day*) OR (illness N1 day*) OR (recovery N1 function*) OR presenteeism OR (sickness N1 absence) OR (work N1 absenteeism) OR (work N1 day N1 loss) OR (work N1 time N1 loss) OR (work N1 participation) OR (occupational N1 health)) OR SU (((repetitive OR repeated OR patterned) N1 absent*) OR (return N1 work) OR (work* N1 capacity) OR (work* N1 disability) OR (work* N1 incapacity) OR (work* N1 incapability) OR (work* N1 inhibition) OR ((medical OR Sick OR disability) N1 leave) OR absente* OR (work N1 (absence OR presence)) OR (disability N1 absence) OR convalescen* OR (sick N1 day*) OR (illness N1 day*) OR (recovery N1 function*) OR presenteeism OR (sickness N1 absence) OR (work N1 absenteeism) OR (work N1 day N1 loss) OR (work N1 time N1 loss) OR (work N1 participation) OR (occupational N1 health))

*S4. Common mental health*

DE "EMPLOYMENT of people with mental illness" OR TI (Common N1 mental N1 (disorder* OR Health OR illness* OR diseas* OR problem*)) OR SU (Common N1 mental N1 (disorder* OR Health OR illness* OR diseas* OR problem*)) OR AB (Common N1 mental N1 (disorder* OR Health OR illness* OR diseas* OR problem*))

*S5. Depression*

TI (Depress* OR Melanchol* OR (Emotional N1 Lability) OR (Seasonal N1 Affective N1 Disorder) OR (Dysthymic N1 Disorder)) OR AB (Depress* OR Melanchol* OR (Emotional N1 Lability) OR (Seasonal N1 Affective N1 Disorder) OR (Dysthymic N1 Disorder)) OR SU (Depress* OR Melanchol* OR (Emotional N1 Lability) OR (Seasonal N1 Affective N1 Disorder) OR (Dysthymic N1 Disorder))

*S6. Anxiety*

TI (anxiet* OR anxious* OR phobi* OR (panic N1 (disorder* OR attack)) OR fear* OR scared OR afraid OR Worrie* OR worry OR Neuros* OR neurotic OR angst OR apprehension OR disquiet* OR dread* OR terrify OR terrified* OR fright* OR horror* OR nervousness OR jitter* OR uneas* OR restlessness* OR (Neurotic N1 Disorder*) OR Psychoneuroses OR (Obsessive N1 Compulsive N1 (Neuros* OR disorder)) OR Panic* OR Claustrophobia* OR (Mental N1 suffering) OR (Negative N1 (feeling* OR emotion OR thought*)) OR (Emotional N1 well N1 being) OR (Emotional N1 (benefit* OR respons*)) OR antianxiet* OR antifear*) OR SU (anxiet* OR anxious* OR phobi* OR (panic N1 (disorder* OR attack)) OR fear* OR scared OR afraid OR Worrie* OR worry OR Neuros* OR neurotic OR angst OR apprehension OR disquiet* OR dread* OR terrify OR terrified* OR fright* OR horror* OR nervousness OR jitter* OR uneas* OR restlessness* OR (Neurotic N1 Disorder*) OR Psychoneuroses OR (Obsessive N1 Compulsive N1 (Neuros* OR disorder)) OR Panic* OR Claustrophobia* OR (Mental N1 suffering) OR (Negative N1 (feeling* OR emotion OR thought*)) OR (Emotional N1 well N1 being) OR (Emotional N1 (benefit* OR respons*)) OR antianxiet* OR antifear*) OR AB (anxiet* OR anxious* OR phobi* OR (panic N1 (disorder* OR attack)) OR fear* OR scared OR afraid OR Worrie* OR worry OR Neuros* OR neurotic OR angst OR apprehension OR disquiet* OR dread* OR terrify OR terrified* OR fright* OR horror* OR nervousness OR jitter* OR uneas* OR restlessness* OR (Neurotic N1 Disorder*) OR Psychoneuroses OR (Obsessive N1 Compulsive N1 (Neuros* OR disorder)) OR Panic* OR Claustrophobia* OR (Mental N1 suffering) OR (Negative N1 (feeling* OR emotion OR thought*)) OR (Emotional N1 well N1 being) OR (Emotional N1 (benefit* OR respons*)) OR antianxiet* OR antifear*)

*S7. Stress*

DE "JOB stress" OR DE "PSYCHOLOGICAL burnout" OR TI (stress* OR Nervousnes* OR (psychological N1 distress) OR (adjustment N1 disorder) OR ((mental OR psycholog*) N1 suffer*) OR burnout) OR AB (stress* OR Nervousnes* OR (psychological N1 distress) OR (adjustment N1 disorder) OR ((mental OR psycholog*) N1 suffer*) OR burnout) OR SU (stress* OR Nervousnes* OR (psychological N1 distress) OR (adjustment N1 disorder) OR ((mental OR psycholog*) N1 suffer*) OR burnout)

*S8*

S4 OR S5 OR S6 OR S7

*S9*

S8 AND S1 AND S2 AND S3

**Appendix 2: quality assessment**

| **Publication** | **Study category** | **1ͥ** | **2** | **3** | **4** | **5** | **6** | **7** | **Total score** | **Quality ͥ ͥ** |
| --- | --- | --- | --- | --- | --- | --- | --- | --- | --- | --- |
| Arends et al. 2014 [1] | Quantitative descriptive | Y ͥ ͥ ͥ | Y | Y | Y | Y | Y | Y | 7 | High |
| Arends et al. 2014 [2] | Quantitative RCT | Y | Y | Y | N ͥ ͥ ͥ ͥ | Y | Y | Y | 6 | High |
| Arends et al. 2014 [3] | Quantitative RCT | Y | Y | Y | Y | Y | Y | Y | 7 | High |
| Endo et al. 2015 [4] | Quantitative non-randomized | Y | Y | Y | Y | Y | Y | Y | 7 | High |
| Ervasti et. Al 2013 [5] | Quantitative non-randomized | Y | Y | Y | Y | Y | Y | Y | 7 | High |
| Ervasti et al. 2014 [6] | Quantitative non-randomized | Y | Y | Y | Y | Y | Y | Y | 7 | High |
| Gaspar et al. 2018 [7] | Quantitative non-randomized | Y | Y | N | Y | Y | C ͥ ͥ ͥ ͥ ͥ | Y | 5 | Moderate |
| Klink, v.d. et al. 2003 [8] | Quantitative RCT | Y | Y | Y | Y | Y | Y | C | 6 | High |
| Koopmans et al. 2010 [9] | Quantitative descriptive | Y | Y | Y | N | Y | Y | Y | 6 | High |
| Koopmans et al. 2011 [10] | Quantitative non-randomized | Y | Y | Y | Y | Y | Y | Y | 7 | High |
| Mather et al. 2019 [11] | Quantitative non-randomized | Y | Y | Y | Y | Y | N | Y | 6 | High |
| Mattila-Holappa 2017 [12] | Quantitative non-randomized | Y | Y | Y | Y | Y | Y | Y | 7 | High |
| Norder 2012 [13] | Qualitative studies | Y | Y | Y | Y | C | Y | Y | 6 | High |
| Norder et al. 2015 [14] | Quantitative descriptive | Y | Y | Y | N | Y | Y | Y | 6 | High |
| Norder et al. 2015 [15] | Quantitative descriptive | Y | Y | Y | Y | Y | Y | N | 6 | High |
| Norder et al. 2015 [16] | Quantitative descriptive | Y | Y | Y | Y | Y | Y | Y | 7 | High |
| Real et al. 2016 [17] | Quantitative descriptive | Y | Y | Y | Y | N | Y | Y | 6 | High |
| Rhenen, v. et al. 2007 [18] | Quantitative RCT | Y | Y | C | Y | Y | C | C | 4 | Moderate |
| Roelen et al. 2010 [19] | Quantitative descriptive | Y | Y | Y | Y | Y | Y | Y | 7 | High |
| Virtanen et al. 2011 [20] | Quantitative non-randomized | Y | Y | Y | Y | Y | N | Y | 6 | High |
| ͥ Answer question 1 etc.  ͥ ͥ high=6-7, moderate=4-5, low=1-3  ͥ ͥ ͥ Yes  ͥ ͥ ͥ ͥ No  ͥ ͥ ͥ ͥ ͥ Can’t tell | | | | | | | | | | |

**Appendix 3: results of the evidence grading**

| **Factor** | **Result** | **Evidence grading** | **Quality assessment** |
| --- | --- | --- | --- |
| SES | *Ervasti et al.[5]*: a lower occupational position increases the risk of RSA  *Koopmans et al.* [10]: man and women with a lower salary had a higher risk of RSA  *Mattila-Hollapa et al.* [12]: employees aged 21-50 with lower education had more often RSA; employees aged 21-34 with a low occupational position had a higher risk of RSA  *Roelen et al.* [19]: employees in lower salary scales had a higher risk of RSA  *Virtanen et al.* [20]: lower occupational grade increases the risk of RSA | Strong (5 studies in the same direction) | High (n=5) |
| Previous SA | *Gaspar et al.* [7]: previous non-CMD SA had a higher rate of RSA  *Koopmans et al.* [9]: the risk of RSA increases after SA due to CMD  *Mather et al.* [11]: the risk of RSA increases after SA due to CMD  *Real et al.* [17]: the probability of RSA was almost double after short-term SA; RSA was associated with SA that ended due to improvement | Strong (4 in the same direction) | High (n=3)  Moderate (n=1) |
| Transport and communication sector | *Gaspar et al.* [7]: employees in the transportation and communication industries had a higher risk of RSA  *Koopmans et al*. [10]: employees in the telecommunication industry had a higher risk of RSA than employees in the post industry  *Real et al.* [17]: employees in the public administration and transport had a higher risk of RSA | Strong (3 in the same direction) | High (n=2)  Moderate (n=1) |
| Age | *Gaspar et al.* [7]: being younger is a risk factor for RSA related to CMD  *Mattila-Hollapa et al* [12]: employees over 34 years of age experienced more often RSA than employees under 34 of age  *Norder et al.* [14]: office and production workers over 55 years of age were at risk of RSA due to mental problems | Weak (3 studies, 2 in same direction, 1 in opposite direction) | High (n=2)  Moderate (n=1) |
| Comorbidity | *Arends et al.* [1]: reporting ≥ 1 chronic diseases decreases the risk of RSA  *Gaspar et al.* [7]: specific comorbidities increases the risk of RSA  *Norder et al.* [13]: comorbid DSM (axis I and II) disorders and comorbid anxiety affect RSA negatively | Weak (3 studies, 2 in same direction, 1 in opposite direction) | High (n=2)  Moderate (n=1) |
| Depression | *Gapar et al.* [7]: depressive disorder had a higher risk of RSA than adjustment and anxiety disorders  *Koopmans et al.* [10]: in men, depressive symptoms were related to a higher RSA than distress symptoms and adjustment disorders. | Weak (2 in the same direction) | High (n=1)  Moderate (n=1) |
| Cognitive intervention | *Arends et al.* [2]: lower incidence of RSA in intervention group  *Van der Klink et al.* [8]: lower mean incidence of RSA in intervention group  *Van Rhenen et al.* [18]: median time to onset of RSA decreased in intervention group | Weak (3 studies, 2 in same direction, 1 in opposite direction) | High (n=2)  Moderate (n=1) |
| High psychological work demands | *Endo et al.* [4]: higher organizational job demands is a risk factor for RSA due to depression  *Norder et al.* [13]: psychological job demands affects RSA negatively | Weak (2 in the same direction) | High (n=2) |
| Tenure | *Koopmans et al.* [10]: women with a job tenure of < 5 years had a higher risk of RSA than women with a job tenure of ≥ 20 years  *Roelen et al.* [19]: employees with a job tenure of < 5 years had a higher risk of RSA than employees with a job tenure of ≥ 5 years; RSA were more frequent in unskilled employees with a short job tenure | Weak (2 in the same direction) | High (n=2) |
| Gender | *Gaspar et al.* [7]: being female is a risk factor for RSA related to CMD  *Koopmans et al.* [9]: no gender differences in RSA due to CMD | Inconsistent (2 in different directions) | High (n=1)  Moderate (n=1) |
| Type of contract | *Ervasti et al.* [6]: temporary employment was not associated with RSA  *Gaspar et al.* [7]: being salaried and being an hourly employees increase the risk of RSA | Inconsistent (2 in different directions) | High (n=1)  Moderate (n=1) |
| Women aged < 45 | *Koopmans et al.* [9]: RSA were more frequent in women aged < 45  *Koopmans et al.* [10]: women aged < 45 had a higher risk of RSA | Inconsistent (2 studies using the same database) | High (n=2) |
| Low supervisor social support | *Arends et al.* [1]: low supervisor social support increased the risk of RSA in the control group | Insufficient (found in 1 study) | High (n=1) |
| Conflict with supervisor | *Arends et al.* [1]: conflict with supervisor increased the risk of RSA (in intervention and control group) | Insufficient (found in 1 study) | High (n=1) |
| Company size | *Arends et al.* [1]: company size of > 100 workers increased the risk of RSA | Insufficient (found in 1 study) | High (n=1) |
| Focus on whether help was needed | *Arends et al.* [3]: employees who focused on whether help was needed with solving problems in the SHARP-at work intervention, had a lower risk on RSA | Insufficient (found in 1 study) | High (n=1) |
| Focus on problems | *Arends et al.* [3]: employees focused on problems in the SHARP-at work intervention, had a higher risk of RSA | Insufficient (found in 1 study) | High (n=1) |
| Being in an union | *Gaspar et al.* [7]: being in a union was a predictor of RSA | Insufficient (found in 1 study) | Moderate (n=1) |
| Inpatient-stays during index leave | *Gaspar et al.* [7]: inpatient-stays during the index leave were not associated with RSA | Insufficient (found in 1 study) | Moderate (n=1) |
| Utilities industry | *Gaspar et al.* [7]: employees in utilities industries had a higher risk of RSA | Insufficient (found in 1 study) | Moderate (n=1) |
| Point-of-service insurance coverage | *Gaspar et al.* [7]: point-of-service insurance coverage was a predictor of RSA | Insufficient (found in 1 study) | Moderate (n=1) |
| Population density | *Gaspar et al.* [7]: population density was a risk factor for RSA due to depression | Insufficient (found in 1 study) | Moderate (n=1) |
| Men and age | *Koopmans et al.* [9]: in men, there was no difference in RSA by age | Insufficient (found in 1 study) | High (n=1) |
| Men aged 45-55 | *Koopmans et al.* [10]: in men, aged 45-55 had a higher risk of RSA | Insufficient (found in 1 study) | High (n=1) |
| Married women | *Koopmans et al*. [10]: married women had a higher risk of RSA than unmarried women | Insufficient (found in 1 study) | High (n=1) |
| Men and marital status | *Koopmans et al.* [10]: no difference in risk of RSA according to marital status in men | Insufficient (found in 1 study) | High (n=1) |
| Familial factors | *Mather et al.* [11]: familial factors do not influence RSA | Insufficient (found in 1 study) | High (n=1) |
| Work dysfunctioning | *Norder et al.* [13]: work dysfunctioning was a predictor of RSA due to depression | Insufficient (found in 1 study) | High (n=1) |
| Stressful work events | *Norder et al.* [13]: stressful work events were a predictor of RSA due to depression | Insufficient (found in 1 study) | High (n=1) |
| Commitment to work | *Norder et al.* [13]: commitment to work was a predictor of RSA due to depression | Insufficient (found in 1 study) | High (n=1) |
| Lifetime numbers of depressive episodes | *Norder et al.* [13]: lifetime number of depressive episode were a predictor of RSA due to depression | Insufficient (found in 1 study) | High (n=1) |
| Substance abuse | *Norder et al.* [13]: substance abuse was a predictor of RSA due to depression | Insufficient (found in 1 study) | High (n=1) |
| Social dysfunctioning | *Norder et al.* [13]: social dysfunctioning was a predictor of RSA due to depression | Insufficient (found in 1 study) | High (n=1) |
| Residual symptoms of depression | *Norder et al.* [13]: residual symptoms of depression were a predictor of RSA due to depression | Insufficient (found in 1 study) | High (n=1) |
| Stressful life events | *Norder et al.* [13]: stressful life events were a predictor of RSA due to depression | Insufficient (found in 1 study) | High (n=1) |
| Severity of first depressive episode | *Norder et al.* [13]: severity of first depressive episode was a predictor of RSA due to depression | Insufficient (found in 1 study) | High (n=1) |
| Severity of last depressive episode | *Norder et al.* [13]: severity of last depressive episode was a predictor of RSA due to depression | Insufficient (found in 1 study) | High (n=1) |
| Duration of last depressive episode | *Norder et al.* [13]: duration of last depressive episode was a predictor of RSA due to depression | Insufficient (found in 1 study) | High (n=1) |
| Duration of first depressive episode | *Norder et al.* [13]: duration of first depressive episode was a predictor of RSA due to depression | Insufficient (found in 1 study) | High (n=1) |
| Age at first episode of depression | *Norder et al.* [13]: age at first episode of depression was a predictor of RSA due to depression | Insufficient (found in 1 study) | High (n=1) |
| High demands – low control | *Norder et al.* [13]: high demands – low control was a predictor of RSA due to depression | Insufficient (found in 1 study) | High (n=1) |
| Effort-reward imbalance | *Norder et al.* [13]: effort-reward imbalance was a predictor of RSA due to depression | Insufficient (found in 1 study) | High (n=1) |
| Decision latitude | *Norder et al.* [13]: decision latitude was a predictor of RSA due to depression | Insufficient (found in 1 study) | High (n=1) |
| Neuroticism | *Norder et al.* [13]: neuroticism was a predictor of RSA due to depression | Insufficient (found in 1 study) | High (n=1) |
| Production workers with emotional disturbance | *Norder et al.* [14]: SA due to emotional disturbance recurred more often in production workers than in office workers | Insufficient (found in 1 study) | High (n=1) |
| Shift work | *Norder et al.* [15]: shift workers had no increased risk of RSA compared with day workers | Insufficient (found in 1 study) | High (n=1) |
| ICD-10 diagnose | *Norder et al.* [16]: RSA’s did not differ across ICD-10 diagnostic categories | Insufficient (found in 1 study) | High (n=1) |
| Public administration sector | *Real et al.* [17]: employees in the administration sector had a higher risk of RSA | Insufficient (found in 1 study) | High (n=1) |
| Aged 30-50 and covered by general scheme | *Real et al.* [17]: aged 30-50 and covered by the general scheme had a higher risk of RSA than aged 30-50 and being self-employed | Insufficient (found in 1 study) | High (n=1) |
| Aged < 31 and self-employed | *Real et al.* [17]: aged < 31 and being self-employed had a higher risk or RSA than aged < 31 and covered by the general scheme | Insufficient (found in 1 study) | High (n=1) |

**References**

1. Arends I, van der Klink JJ, Van Rhenen W, de Boer MR, Bültmann U. Predictors of recurrent sickness absence among workers having returned to work after sickness absence due to common mental disorders. Scandinavian journal of work, environment & health. 2014:195-202; https://doi:10.5271/sjweh.3384.

2. Arends I, van der Klink JJ, van Rhenen W, de Boer MR, Bültmann U. Prevention of recurrent sickness absence in workers with common mental disorders: results of a cluster-randomised controlled trial. Occupational and environmental medicine. 2014;71(1):21-9; https://doi.org/10.1136/oemed-2013-101412.

3. Arends I, Bültmann U, Nielsen K, van Rhenen W, de Boer MR, van der Klink JJ. Process evaluation of a problem solving intervention to prevent recurrent sickness absence in workers with common mental disorders. Social Science & Medicine. 2014;100:123-32; https://doi.org/10.1016/j.socscimed.2013.10.041.

4. Endo M, Muto T, Haruyama Y, Yuhara M, Sairenchi T, Kato R. Risk factors of recurrent sickness absence due to depression: a two-year cohort study among Japanese employees. International archives of occupational and environmental health. 2015;88:75-83; https://doi.org/10.1007/s00420-014-0939-4.

5. Ervasti J, Vahtera J, Pentti J, Oksanen T, Ahola K, Kivimäki M, et al. Depression-related work disability: socioeconomic inequalities in onset, duration and recurrence. Plos one. 2013;8(11):e79855; https://doi.org/10.1371/journal.pone.0079855.

6. Ervasti J, Vahtera J, Virtanen P, Pentti J, Oksanen T, Ahola K, et al. Is temporary employment a risk factor for work disability due to depressive disorders and delayed return to work? The Finnish Public Sector Study. Scandinavian journal of work, environment & health. 2014:343-52; https://doi:10.5271/sjweh.3424.

7. Gaspar FW, Zaidel CS, Dewa CS. Rates and predictors of recurrent work disability due to common mental health disorders in the United States. PLoS One. 2018;13(10):e0205170; https://doi.org/10.1371/journal.pone.0205170.

8. van der Klink JJ, Blonk RW, Schene AH, van Dijk FJ. Reducing long term sickness absence by an activating intervention in adjustment disorders: a cluster randomised controlled design. Occupational and environmental medicine. 2003;60(6):429-37; https://doi.org/10.1136/oem.60.6.429.

9. Koopmans PC, Roelen CA, Bültmann U, Hoedeman R, van der Klink JJ, Groothoff JW. Gender and age differences in the recurrence of sickness absence due to common mental disorders: a longitudinal study. BMC Public Health. 2010;10(1):1-9; https://doi.org/10.1186/1471-2458-10-426.

10. Koopmans PC, Bültmann U, Roelen CA, Hoedeman R, van der Klink JJ, Groothoff JW. Recurrence of sickness absence due to common mental disorders. International archives of occupational and environmental health. 2011;84:193-201; https://doi.org/10.1007/s00420-010-0540-4.

11. Mather L, Blom V, Bergström G, Svedberg P. Adverse outcomes of sick leave due to mental disorders: a prospective study of discordant twin pairs. Scandinavian Journal of Public Health. 2019;47(2):127-36; https://doi.org/10.1177/1403494817735755.

12. Mattila-Holappa P, Ervasti J, Joensuu M, Ahola K, Pentti J, Oksanen T, et al. Do predictors of return to work and recurrence of work disability due to mental disorders vary by age? A cohort study. Scandinavian journal of public health. 2017;45(2):178-84; https://doi.org/10.1177/1403494816686467.

13. Norder G, Roelen CA, van Rhenen W, Buitenhuis J, Bültmann U, Anema JR. Predictors of recurrent sickness absence due to depressive disorders–A Delphi approach involving scientists and physicians. PloS one. 2012;7(12):e51792; https://doi.org/10.1371/journal.pone.0051792.

14. Norder G, Bültmann U, Hoedeman R, Bruin Jd, van der Klink JJ, Roelen CA. Recovery and recurrence of mental sickness absence among production and office workers in the industrial sector. The European Journal of Public Health. 2015;25(3):419-23; https://doi.org/10.1093/eurpub/cku202.

15. Norder G, Roelen CA, Bültmann U, van der Klink JJ. Shift work and mental health sickness absence: a 10-year observational cohort study among male production workers. Scandinavian journal of work, environment & health. 2015:413-6; https://doi:10.5271/sjweh.3501.

16. Norder G, Hoedeman R, De Bruin J, Van Rhenen W, Roelen C. Time to recurrence of mental health-related absence from work. Occupational Medicine. 2015;65(7):574-7; https://doi.org/10.1093/occmed/kqv109.

17. Real E, Jover L, Verdaguer R, Griera A, Segalàs C, Alonso P, et al. Factors associated with long-term sickness absence due to mental disorders: a cohort study of 7.112 patients during the Spanish economic crisis. PLoS One. 2016;11(1):e0146382; https://doi.org/10.1371/journal.pone.0146382.

18. van Rhenen W, Blonk RW, Schaufeli WB, van Dijk FJ. Can sickness absence be reduced by stress reduction programs: on the effectiveness of two approaches. International archives of occupational and environmental health. 2007;80:505-15; https://doi.org/10.1007/s00420-006-0157-9.

19. Roelen C, Koopmans P, Anema J, Van Der Beek A. Recurrence of medically certified sickness absence according to diagnosis: a sickness absence register study. Journal of occupational rehabilitation. 2010;20:113-21; https://doi.org/10.1007/s10926-009-9226-8.

20. Virtanen M, Kawachi I, Oksanen T, Salo P, Tuisku K, Pulkki-Råback L, et al. Socio-economic differences in long-term psychiatric work disability: prospective cohort study of onset, recovery and recurrence. Occupational and Environmental Medicine. 2011;68(11):791-8; https://doi.org/10.1136/oem.2010.061101.
